# Limited Visibility Aware Motion Planning for Autonomous Valet Parking Using Reachable Set Estimation

**DOI:** 10.3390/s21041520

**Published:** 2021-02-22

**Authors:** Seongjin Lee, Wonteak Lim, Myoungho Sunwoo, Kichun Jo

**Affiliations:** 1 Department of Automotive Engineering, Hanyang University, 222 Wangsimni-ro, Seongdong-gu, Seoul 04763, Korea; seongjin90@gmail.com (S.L.); lwt1849@gmail.com (W.L.); msunwoo@hanyang.ac.kr (M.S.); 2 Department of Smart Vehicle Engineering, Konkuk University, Seoul 05029, Korea

**Keywords:** automated valet parking, planning under uncertainty, replanning, utility theory

## Abstract

Autonomous driving helps drivers avoid paying attention to keeping to a lane or keeping a distance from the vehicle ahead. However, the autonomous driving is limited by the need to park upon the completion of driving. In this sense, automated valet parking (AVP) system is one of the promising technologies for enabling drivers to free themselves from the burden of parking. Nevertheless, the driver must continuously monitor the automated system in the current automation level. The main reason for monitoring the automation system is due to the limited sensor range and occlusions. For safety reasons, the current field of view must be taken into account, as well as to ensure comfort and to avoid unexpected and harsh reactions. Unfortunately, due to parked vehicles and structures, the field of view in a parking lot is not sufficient for considering new obstacles coming out of occluded areas. To solve this problem, we propose a method that estimates the risks for unobservable obstacles by considering worst-case assumptions. With this method, we can ensure to not act overcautiously while moving safe. As a result, the proposed method can be a proactive approach to consider the limited visibility encountered in a parking lot. In the proposed method, occlusion can be efficiently reflected in the planning process. The potential of the proposed method is evaluated in a variety of simulations.

## 1. Introduction

Autonomous driving technology is being used to support the automotive industry in several ways, ranging from safety concerns to driving comfort. Advances in technology have eliminated drivers’ need to pay attention to keeping to driving lanes or maintaining the distance between cars while driving. However, the convenience of automation is limited by the need to park upon the completion of driving, which distresses drivers both mentally and physically [1]. Unfortunately, 23% of all traffic accidents occur in parking lots (car to car collision and car to pedestrian collision), of which 30% are due to parking in an occluded area, where serious injury and damage can occur [2]. If parking can be conducted automatically without human intervention, such a system has the potential to make the driver more comfortable and safer [3].

In this sense, automated valet parking (AVP) systems are one of the most promising technologies for enabling drivers to free themselves from the burden of parking. In 2003, the first automated parking system was introduced into the automotive market [4], which could steer itself into a parking spot. Currently, this initial system has been extended to automated valet parking (AVP), which allows a driver to call a car by pressing a button or instructs the car to park on its own. In the level of automation currently available; however, the driver must continuously monitor the system due to safety concerns.

The main reason for monitoring the automation system is due to the limited sensor range based on the measurement principle, adverse environmental conditions, or occlusions. In addition, for safety reasons, the current field of view must be taken into account, as well as to ensure comfort and to avoid unexpected and harsh reactions. Unfortunately, due to parked vehicles and other structures, the current field of view for sensors in a parking lot is not sufficient for considering new obstacles (pedestrians) coming out of occluded areas. In many approaches of dealing with limited sensor range, new obstacles are treated by reactive planning [5,6,7,8,9,10,11], which only deals with visible obstacles. However, if the visibility is limited, proactive planning is required, to detect risks inside an otherwise invisible area.

To reflect the invisible area, we propose a proactive approach to overcome the limited visibility encountered in a parking lot. We divided the proposed approach into three steps: Potential Collision Boundary Estimation, Reachable Set Estimation, and Planning in Limited Visibility. From the first to second step, we estimate the probable maneuver alternatives of other participants by modeling reachable states limited by physics. Then, we introduce a methodology to stay collision-free while considering an environment that includes both limited visibility and possible unexpected behaviors in the last step.

The main contributions are:

1. a deterministic method to represent the risks for unobservable obstacles
2. a framework for generating a safe speed profile in conditions of limited visibility

Previous approaches for the probabilistic modeling of occlusions could not guarantee safe driving; however, we had the planner use the collision risk in an optimized manner by modeling deterministic collision risks in a parking lot. In addition, the problem of occlusion in a parking lot has not yet been covered in other papers. Here, we guarantee the safety issues in conditions of limited visibility by using a deterministic risk estimation process.

The remainder of this paper is structured as follows: In the next section, we shortly review the related work. In Section 3, we start with an overview of the proposed method, before presenting our new approach in Section 4 and Section 5. In Section 6, we evaluate the approach in a simulation. Finally, we conclude our work in Section 7.

## 2. Related Works

Safe motion planning requires the consideration of limited visibility from invisible areas in the environment. Many works have addressed different aspects of risk assessment and safe planning for conditions of limited visibility. Some researchers have studied the probabilistic risk assessment of occluded areas [12,13,14,15]. One of them [12] proposed a probabilistic risk assessment method using the volume of traffic on the road, whereas [13] addressed a similar approach using a damage model based on the masses and velocities of two vehicles. However, these algorithms have limitations in that they require the volume of traffic and the mass of the vehicle, which cannot be measured from a sensor. Another method presents the threat level as a probability distribution, using time-to-entry (TTE) and a Bayesian network [14]. In addition, ref. [15] proposed a graphical model capable of describing the risk of a road segment over time, and then addressed the occupancy estimation using a dynamic Bayesian network. Even though these studies explicitly represent the risk as a probability such that the risk can be considered in the planning process, expressing the risk as a probability cannot ensure a provable safety.

To remove the uncertainty of probability, many works use a deterministic approach that solves a worst-case problem [16,17,18,19,20,21,22]. The planner presented in [16] dealt with uncertainty predictions at intersections and considers emergency braking before reaching the intersection. The work; however, did not consider sensor range or vehicles approaching behind the perception field. To plan for a fail-safe motion, ref [17] evaluated the occupancy set of vehicles in the environment and considered the existence of an emergency maneuver, e.g., a lane change. This work did not take the perceptive field of the vehicle into account. In another study, ref. [18] presented a method to prevent potentially hazardous situations by with the car cautiously advance into the intersection while regarding possibly occluded traffic participants using a dynamic grid map. Ref. [19] proposed a method to analyze the safety of a given trajectory with respect to occlusions. Ref. [20] focused on motion planning given an uncertain environment model with occlusions. They presented a method for remaining collision-free for the worst-case evolution of a given scene. Refs. [21,22] formalized the potential risk due to occlusions and limited sensor capability by over-approximating all possible states of unobservable obstacles using state intervals. However, these approaches cannot be applied to valet parking scenarios. Since these algorithms assume that the obstacles pop out according to the topology of the road map, they cannot cautiously assess the spaces between parked vehicles.

## 3. Overview

**Figure 1:**
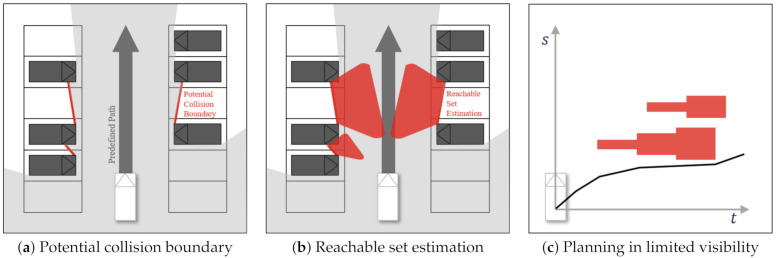
An overall architecture of limited visibility aware motion planning.

The overall process is described in Figure 1a–c. We assume that a path is generated by a priori planner. In other words, we focus on how to consider unobservable obstacles in the speed planning process.

The first step is to estimate a potential collision boundary, which is a boundary from which unobservable obstacles may pop out from, as shown in Figure 1a. By comparing the range sensor and object data, we calculate the location of the potential collision boundary described as the red lines in Figure 1a. Then, we assume that the unobservable obstacles may pop out from these potential collision boundaries.

Unobservable obstacles can be predicted by using the reachable set over-approximations introduced by [23]. A reachable set refers to a method for calculating the distance an obstacle will reach if it is in the collision area, as shown in Figure 1b. We use a simple constant velocity model to predict the reachable set.

The planner plans the speed profile for the unobservable obstacles by defining the problem in the distance-time domain described in Figure 1c. To define this problem, we calculate the start and end times of intersecting unobservable obstacles while following a predefined path. Then, the planner generates a speed profile by solving the A* search algorithm [24]. The details will be explained in the following sections.

## 4. Risk in Limited Visibility

When the surrounding environment is detected by a sensor mounted on an autonomous vehicle, the field of view might be partially occluded by obstacles such as parked vehicles or constructions. In addition, there may be unobservable pedestrians in the parking lot, which have the potential to collide with the ego vehicle. Therefore, a method for considering occluded areas must be formulated for safe driving. The surrounding environment of the ego vehicle is divided to areas according to whether the vehicle can detect when an obstacle exists. The area surrounded by observable obstacles is known as a “free space”. Conversely, areas with obstacles that interfere with detection of obstacles are called “unknown areas”. A collision boundary is the border between the free space and the unknown area. Among the collision boundaries, the boundary where unobservable obstacles may pop out is called the “potential collision boundary (PCB)”. We assume here that the unobservable risk from limited visibility originates from the potential collision boundary. The details for calculating PCB will be explained in the following section.

### 4.1. Estimating the Potential Collision Boundary

To estimate the risk in the occluded boundary, it is first necessary to define a mathematical model for the potential collision boundary (PCB). This model can be derived in three steps. First, candidate points are extracted from the range sensor. Then, based on the extracted candidate points, the free space boundary is formulated as a polygon set. Finally, by subtracting points from observable obstacles at the free space boundary, the PCB can be defined.

**Figure 2:**
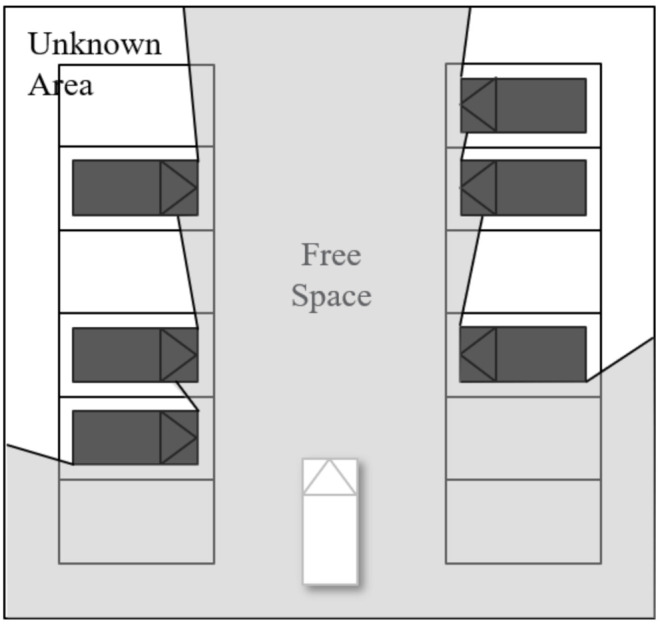
Area classified by the occlusion boundary.

In this paper, we use LiDAR sensor to detect the surrounding environment of the ego vehicle. LiDAR provides a point cloud as raw data. Since the point cloud follows the ego vehicle’s field of view (FoV), there is no obstacle between the ego vehicle and the point cloud. Therefore, an area consisting of lines that connect each point cloud can be considered a free space, as shown by the gray area in Figure 2.

**Figure 3:**
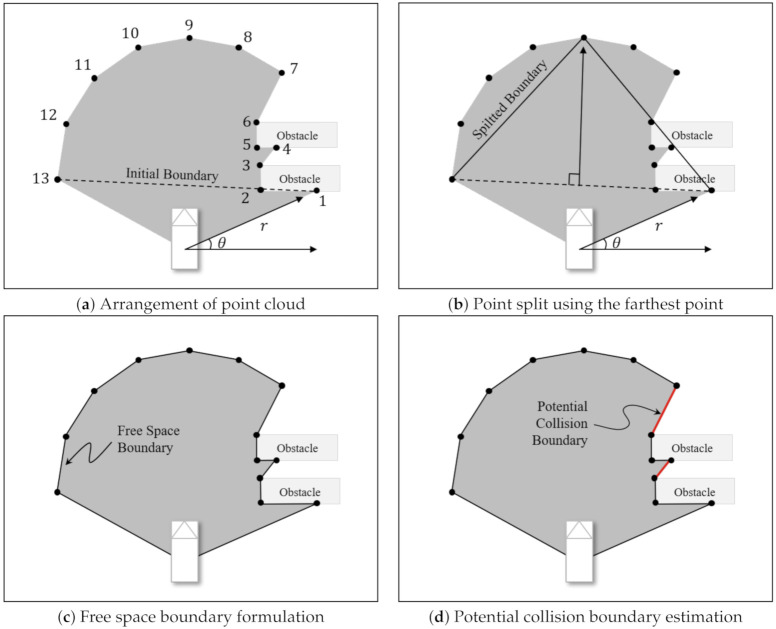
Steps for estimating the potential collision boundary based on candidate points.

The set of free space polygon is approximated using a line segmentation method. First, the cloud points are arranged in the order of vehicle center and angle, as shown in Figure 3a. Then, we generate a straight line that passes through the two points with the biggest difference in angle, as shown in Figure 3b. Next, we calculate the distances between the straight line and the point from the point cloud to find the farthest point. If the farthest distance is larger than a threshold, the method divides the line at that point. This finding and dividing process is repeated until the farthest distance is less than the threshold. Finally, the free space boundary can be formulated as a set of line segments by adding lines connecting the divided points (Figure 3c). A potential collision may occur between the obstacles (points 3 to 4) and between the obstacle and the maximum range of the sensor (points 6 to 7). For this reason, the cloud points for obstacles (points 1 to 6) are subtracted from the free space boundary, except for the first and the last cloud points of the obstacles. Through the above process, the PCB can be obtained as a set of line segments, as shown by the red lines in Figure 3d.

### 4.2. Motion Prediction with Reachable Set Estimation

**Figure 4:**
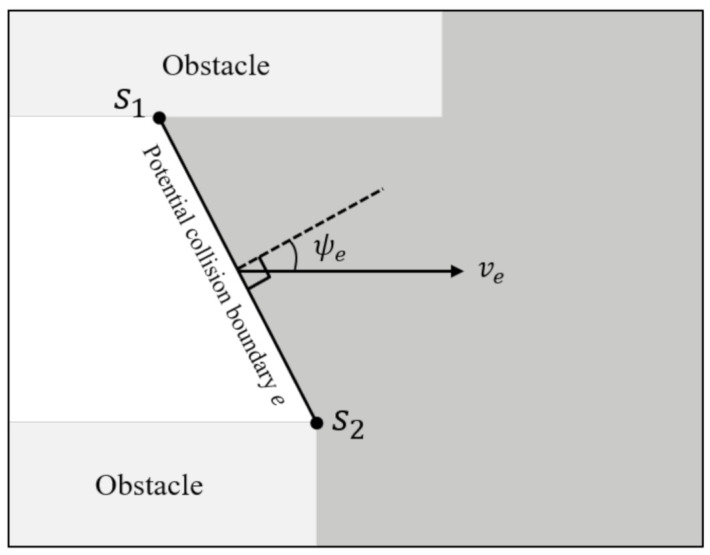
Notations for identifying the potential collision boundary.

To estimate the collision risk, we characterize the potential risks from the potential collision boundary. In addition, the ego vehicle and other obstacles is modeled as rectangular shapes, and over-approximations of the unobservable obstacles are modeled using polygons. Here, we define one unobservable obstacle for each potential collision boundary *e* with the following state, referred as intervals in orientation $\psi_{e}\left(0\right)$, velocity $v_{e}\left(0\right)$, and initial position $s_{e}\left(0\right)$ formulated by the two vertices $s_{1}$ and $s_{2}$ (see Figure 4). This can be formulated as

(1) $$s_{e}\left(0\right)\in \left[\begin{array}{c} \left(\begin{array}{c} s_{1,x}\\ s_{1,y} \end{array}\right),\left(\begin{array}{c} s_{2,x}\\ s_{2,y} \end{array}\right) \end{array}\right]$$

(2) $$\psi_{e}\left(0\right)\in \left[\begin{array}{c} \psi_{min},\psi_{max} \end{array}\right]$$

(3) $$v_{e}\left(0\right)\in \left[\begin{array}{c} v_{min},v_{max} \end{array}\right]$$

The reachable set approximations from [23] for such initial state sets of PCB is derived using the initial state intervals of PCB in Equations (1) to (3). Based on Kamm’s circle [25], these intervals describe the physically reachable area, limited by the absolute possible acceleration. For simplicity, we assume that the state set of PCB can be represented in local coordinates as follows,

(4) $$s_{e}\left(0\right)\in \left[\begin{array}{c} \left(\begin{array}{c} 0\\ 0 \end{array}\right),\left(\begin{array}{c} {\bar{s}}_{x}\\ {\bar{s}}_{y} \end{array}\right) \end{array}\right]$$

(5) $$\psi_{e}\left(0\right)\in \left[\begin{array}{c} -\psi_{max},\psi_{max} \end{array}\right]$$

(6) $$v_{e}\left(0\right)\in \left[\begin{array}{c} \underset{\bar{}}{v},\bar{v}. \end{array}\right]$$

Here, we combine a formulation of Kamm’s circle with center c(t) and radius r(t) and the boundary of the circle over time b(t).

(7) $$c\left(t\right)=\left(\begin{array}{c} s_{x}\left(0\right)\\ s_{y}\left(0\right) \end{array}\right)+\left(\begin{array}{c} v_{x}\left(0\right)\\ v_{y}\left(0\right) \end{array}\right)t$$

(8) $$r\left(t\right)=\frac{1}{2}a_{max}t^{2}$$

(9) $$b_{x}\left(t\right)=v_{0}t-\frac{a_{max}^{2}t^{3}}{2v_{0}}$$

(10) $$b_{y}\left(t\right)=\sqrt{\frac{1}{4}a_{max}^{2}t^{4}-{\left(\frac{a_{max}^{2}t^{3}}{2v_{0}}\right)}^{2}}$$

**Figure 5:**
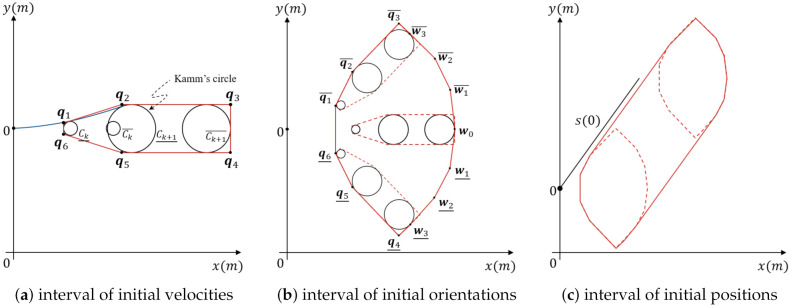
Occupancy over-approximations for initial intervals.

Figure 5a–c describes a representation of this estimation.

#### 4.2.1. Interval of Initial Velocities

Through the interval of the initial velocity $v_{0}\in \left[\underset{\bar{}}{v},\bar{v}\right]$ with known orientation ψ(0)=0, and the initial position $s\left(0\right)={\left(0,0\right)}^{T}$, we can formulate

(11) $$\underset{\bar{}}{c}\left(t\right)=c\left(t,\underset{\bar{}}{v}\right),\bar{c}\left(t\right)=c\left(t,\bar{v}\right)$$

and likewise, $b_{x}$ and $b_{y}$. Here, $\underset{\bar{}}{\cdot }$ is the minimum value of the notation, and $\bar{\cdot }$ refers to the maximum value of the notation. The reachable set of the obstacle for a time period of $\tau_{k}=\left[t_{k},t_{k+1}\right]$ can be approximated by the polygon with the points from $q_{1}$ to $q_{6}$.

(12) $$q_{1}={\left(\underset{\bar{}}{c_{x}}\left(t_{k}\right)-r\left(t_{k}\right),r\left(t_{k}\right)\right)}^{T}$$

(13) $$q_{2}={\left(\underset{\bar{}}{b_{x}}\left(t_{k+1}\right),r\left(t_{k+1}\right)\right)}^{T}$$

(14) $$q_{3}={\left(\bar{c_{x}}\left(t_{k+1}\right)+r\left(t_{k+1}\right),r\left(t_{k+1}\right)\right)}^{T}$$

(15) $$q_{4}={\left(\bar{c_{x}}\left(t_{k+1}\right)-r\left(t_{k+1}\right),-r\left(t_{k+1}\right)\right)}^{T}$$

(16) $$q_{5}={\left(\underset{\bar{}}{b_{x}}\left(t_{k+1}\right),-r\left(t_{k+1}\right)\right)}^{T}$$

(17) $$q_{6}={\left(\underset{\bar{}}{c_{x}}\left(t_{k}\right)-r\left(t_{k}\right),-r\left(t_{k}\right)\right)}^{T}$$

as shown in Figure 5a.

The left side of the equation is coincidence with $O_{1}$, which is the red polygon in Figure 5a, but $q_{3}$ and $q_{4}$ are estimated by using $\bar{v}$. This boundary includes all $v_{i}\in \left[\underset{\bar{}}{v},\bar{v}\right]$, and each circle $C_{k+1}\left(v_{i}\right)$ has the same radius $r(t_{k+1})$. Circle center is bounded as $c_{x}(t_{k+1}\in \left[{\underset{\bar{}}{c}}_{x}\left(t_{k+1}\right),{\bar{c}}_{x}\left(t_{k+1}\right)\right],c_{y}=0$. Therefore, the polygon $P(q_{1},q_{2},q_{3},q_{4},q_{5},q_{6})$ spanned by $\underset{\bar{}}{C_{k}},\underset{\bar{}}{C_{k+1}}$, and $\bar{C_{k+1}}$ is equivalent to $O_{1}$. This polygon contains all $C_{t}\left(v_{i}\right)$ with $t\in [t_{k},t_{k+1}]$, proving that this polygon is an over-approximation of all sets that can be reached for unobservable obstacles with initial velocities

#### 4.2.2. Interval of Initial Orientations

Initial orientation interval $\psi \left(0\right)\in \left[-\psi_{max},\psi_{max}\right]$ rotates the entire reachable set $P(q_{1},q_{2},q_{3},q_{4},q_{5},q_{6})$. We can over-approximate this rotated set. The boundaries of the set is formulated by rotating $q_{1},q_{2},q_{3}$ counterclockwise to $\bar{q_{1}},\bar{q_{2}},\bar{q_{3}}$ and $q_{4},q_{5},q_{6}$ clockwise to $\underset{\bar{}}{q_{4}},\underset{\bar{}}{q_{5}},\underset{\bar{}}{q_{6}}$ using $\psi_{max}$. Furthermore, the furthest longitudinal point $p_{long}={\left(\bar{c_{x}}\left(k+1\right)+r\left(t_{k+1}\right),0\right)}^{T}$ of each circle can be over-approximated by

(18) $$w_{0}={\left(\frac{\bar{c_{x}}+r\left(t_{k+1}\right)}{\cos\frac{\theta }{2}}\right)}^{T},\theta =\frac{\psi_{max}}{n}$$

(19) $$\bar{W_{j}}=R_{\theta_{j}}w_{0}$$

(20) $$\underset{\bar{}}{W_{j}}=-R_{\theta_{j}}w_{0}$$

with j∈[1,n]. An example approximation of the circle with $\psi_{max}$ of around 45deg is achieved with n=3. Figure 5b describes this formula, proving that each rotated polygon over $\left[-\psi_{max},\psi_{max}\right]$ is included by the following polygon.

(21) $$P(\bar{q_{1}},\bar{q_{2}},\bar{q_{3}},{\bar{w}}_{n},\cdots ,{\bar{w}}_{1},w_{0},\underset{\bar{}}{w_{n}},\underset{\bar{}}{q_{4}},\underset{\bar{}}{q_{5}},\underset{\bar{}}{q_{6}})$$

#### 4.2.3. Interval of Initial Positions

The transform due to intervals of initial position is defined by using linear interpolation of the polygon $\underset{\bar{}}{P}$ for $\underset{\bar{}}{s_{(}0)}={\left(0,0\right)}^{T}$ as described in the previous subsection. First, we create a duplicate $\bar{P}$ that is translated by $\bar{s}\left(0\right)={\left(s_{x},s_{y}\right)}^{T}$ and then compute the total polygon of both polygons $O_{1}\left(\tau_{k}\right)=Conv\left(\underset{\bar{}}{P},\bar{P}\right)$. The occupancy of each possible position on the line segment is easily calculated by the linearity of translations and the line segments. Thus, $O_{1}\left(\tau_{k}\right)$ is an over-approximation of the possible reachable set of an unobservable obstacle with a bounded initial state. The resulting over-approximation is described with example parameters in Figure 5c.

The total occupancy $O_{1}$ refers to the reachable area if the unobservable obstacle originates from the potential collision boundary *e*. By applying the reachable set estimation, we can calculate the intersection area between the reachable set and the predefined path. In the planning phase, we generate an optimal speed profile to consider for the intersection area. Further details will be explained in the following chapter.

## 5. Planning in Limited Visibility

Prior to planning the speed profile for conditions of limited visibility, the path should be defined. Since most situations can be formulated as driving along a predefined lane with arbitrary geometry, we assume that the problem of roaming in a parking lot is similar to the problem of driving along a lane. For this reason, we use the trajectory planner defined in [10] to generate a path in a parking lot. In the speed planner, we only calculate in the longitudinal direction, i.e., we set the problem in a one-dimensional direction. This approach is known as a path-velocity decomposition [26].

We first construct the planning environments for the problem as written in [27]. This approach provides a general solution for a longitudinal direction in on-road driving if the planning environments can be described. The planning environments include how to obtain the desired speed, how to represent obstacles, and how to set a goal. Then, the planner solves the optimization problem using A*, which is a well-known planning algorithm.

The two main differences with the previous approach are:

1. the proposed approach considers not only observable obstacles but also unobservable obstacles
2. the proposed approach does not require a topology map

We predict the motion of unobservable obstacles using the over-approximation of PCBs in the previous section. The PCBs are over-approximated by estimating all possible motions when unobservable obstacles suddenly come out from the PCBs, as shown in Figure 5. This over-approximated area covers the position where unobservable obstacles pop out. Therefore, through the over-approximation, unobservable obstacles can be represented as with observability.

In addition, the previous approach can only represent obstacles on the road topology as the planning environments. This is appropriate to on-road planning, but not in parking lots since unobservable obstacles can be a pedestrian and a cyclist who does not follow the road topology. However, the proposed approach can deal with these obstacles. The PCBs are calculated from LiDAR sensor field of view, which is not associated with the road topology, then, the over-approximation of PCBs can cover the unpredictable motion of unobservable obstacles such as a pedestrian or a cyclist.

### 5.1. Problem Statement

Assume that $p_{i}={\left(p_{x},p_{y}\right)}^{T}\in R^{2}$ is a point on the center line of a predefined path *c*, then $s(p_{i})\in R$ denotes the traveled distance along the path in the interval $\left[p_{0},p_{i}\right]$. The velocity is bounded as $\left[0,v_{max}\right]$, and then, $v_{max}\left(s\right)$ is a function of the path’s curvature κ at distance *s*, i.e., $v_{max}\left(s\right)=f\left(\kappa \left(s\right)\right)$. *u*, the acceleration of the vehicle, is the system input within $\left[a_{min},a_{max}\right]$. The longitudinal movement of the vehicle is formulated by the differential equations as follows,

(22) $$\left[\begin{array}{c} \dot{s}\\ \ddot{s} \end{array}\right]=\left[\begin{array}{c} 0\;1\\ 0\;0 \end{array}\right]\left[\begin{array}{c} s\\ \dot{s} \end{array}\right]+\left[\begin{array}{c} 0\\ 1 \end{array}\right]u$$

Along the path *c*, there is a finite set *E* of obstacles $E_{i}$ that intersects with the path for a time period $\tau^{E_{i}}=\left[t_{start}^{E_{i}},t_{end}^{E_{i}}\right]$ at a specific position $s^{E_{i}}\left(t\right)$ and time $\tau^{E_{i}}$. These obstacles should not occupy the position of the ego vehicle $s_{ego}$ at any time. The goal of the planner is to find a valid speed profile. This plan can be derived as an optimization problem over *f*, such that

(23) $$\min_{u(t)}\left(f\right)=\min_{u(t)}f(s_{ego},{\dot{s}}_{ego},{\ddot{s}}_{ego},\kappa \left(s\right),E)$$

This problem must have only one global minimum for various constraints. The optimization problem can be converted to a discrete problem in the state space $X\subseteq R^{3}$ with states $x={\left[s.v.t\right]}^{T}\in X$. Then, this problem can be solved using an A* graph search [24]. The state $x_{i}$ denotes the state at the planning step *i*. We construct the searching graph for A* online by sampling a set of actions A during a time step Δt. At each iteration of its main loop, A* needs to determine which of its paths to extend. It does so based on the cost of the path, using an estimate of the cost required to extend the path to the goal. Specifically, A* selects the path that minimizes

(24) $$f\left(x_{i}\right)=g\left(x_{i}\right)+h\left(x_{i}\right)$$

where g(·) is the cost of the path from the initial state, and h(·) is a heuristic function that estimates the cost for the cheapest path from the current state to the goal. A* terminates when the path it chooses to extend is a path from start to goal, or if there are no paths eligible to be extended. The heuristic function is problem-specific. If the heuristic function is admissible, meaning that it never overestimates the actual cost to get to the goal, A* is guaranteed to return the least-cost path from the start to the goal. The following sections describe how the A* graph is constructed, its cost function, and heuristics.

### 5.2. Transition Model

The discretized transition model can be written as

(25) $$x_{i+1}=\left[\begin{array}{c} s_{i+1}\\ v_{i+1}\\ t_{i+1} \end{array}\right]=\left[\begin{array}{c} 1\;\Delta t\;0\;0\\ 0\;1\;0\;0\\ 0\;0\;1\;\Delta t \end{array}\right]\left[\begin{array}{c} s_{i}\\ v_{i}\\ t_{i}\\ 1 \end{array}\right]+\left[\begin{array}{c} \frac{1}{2}{\left(\Delta t\right)}^{2}\\ \Delta t\\ 0 \end{array}\right]a_{i}$$

where $a_{i}$ refers the action that is selected in the step *i* and expanded for Δt. (25) represents the longitudinal motion of the ego vehicle. The objective of planning is to find a set of $x_{i}$ in an acyclic searching graph. To reduce the computational time, we discretize the action space. By using a discretized action space, the state $x_{i}$ is expanded to reach a goal state $x_{G}$. At that time, the searching graph is generated, then, we select the minimum cost state from among the next states. This process repeats until the state $x_{i}$ reaches $x_{G}$.

### 5.3. Cost Function

$g(x_{i},a,x_{i+1},E)$ is the step cost in state $x_{i}$ to traverse to state $x_{i+1}$ for taking action *a*. The total cost is the cumulative cost of all steps along the path, $\sum_{x_{i}=x_{start}}^{x_{goal}}g(x_{i},a,x_{i+1},E)$. The goal is to find the path from the initial state to a goal state that incurs minimal costs. To represent the different parameters of the optimization problem, a weighted sum of different costs is used as the stop cost.

(26) $$g\left(x_{i},a,x_{i+1},E\right)=\omega_{V}\cdot g_{V}\left(s_{i+1}\right)+\omega_{A}\cdot g_{A}\left(a\right)+\omega_{E}\cdot g_{E}\left(x_{i},x_{i+1},E\right)$$

where $g_{V}\left(x_{i+1}\right)$ refers to the cost for the desired speed, $g_{A}\left(a\right)$ denotes the cost of taking action *a*, and $g_{E}\left(x_{i},x_{i+1},E\right)$ is the cost for a collision during traveling from $x_{i}$ to $x_{i+1}$. In addition, $\omega_{V},\omega_{A}$, and $\omega_{E}$ are the weight factors for the desired speed, acceleration, and collision, respectively. The following sections derive the formulation for each cost function.

#### 5.3.1. Velocity Cost

$v_{des}\left(s\right)$ is a function of the desired speed at position *s* without obstacles. This speed combines the speed limit $v_{law}\left(s\right)$ and $v_{curve}\left(s\right)$ along the path length *s*. $v_{curve}$ is formulated according to a maximum allowed lateral acceleration $a_{lat,curve}$ in the curvature radius $r_{curve}\left(s\right)$ written in [28].

(27) $$v_{curve}\left(s\right)=\sqrt{a_{lat,curve}r_{curve}\left(s\right)}$$

**Figure 6:**
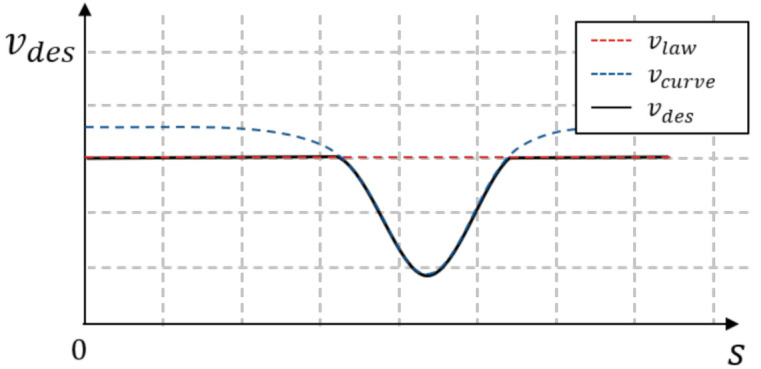
Along the path, the velocity is limited by the speed limit and the curvature. The curve approach velocity is part of the desired speed.

Then, the desired speed is the minimum of $v_{law}$ and $v_{curve}$ as shown in Figure 6. We set the speed limit in a parking lot to be under 15 km/h, and the maximum allowed lateral acceleration is 2 m/s ${}^{2}$.

The velocity cost $g_{V}$ is derived as difference to the desired speed $v_{des}$. A too high speed is punished quadratically, while a too low speed is punished linearly to allow for a lower speed when decelerating upon obstacles. Here, $g_{V}\left(x_{i+1}\right)$ can then be written as follows:

(28) $$g_{V}\left(x_{i+1}\right)=\left\{\begin{array}{cc} {\left(v_{i+1}-v_{des}\left(s_{i+1}\right)\right)}^{2},\; & v_{i+1}>v_{des}\left(s_{i+1}\right)\\ 0,\; & v_{i+1}=v_{des}\left(s_{i+1}\right)\\ \frac{1}{2}{\left(v_{des}\left(s_{i+1}\right)-v_{des}\right)}^{2},\; & v_{i+1}<v_{des}\left(s_{i+1}\right) \end{array}\right.$$

#### 5.3.2. Acceleration Cost

The cost of taking action depends on the acceleration value *a*. The objective of the acceleration cost is to punish either a too high or too low acceleration, and to maintain the current acceleration. Here, $g_{A}\left(a\right)$ is written as follows:

(29) $$g_{A}\left(a\right)=\frac{1}{2}a^{2}$$

The use of an acceleration cost or a high value of acceleration weighting factor allows for comfortable driving.

#### 5.3.3. Collision Cost

**Figure 7:**
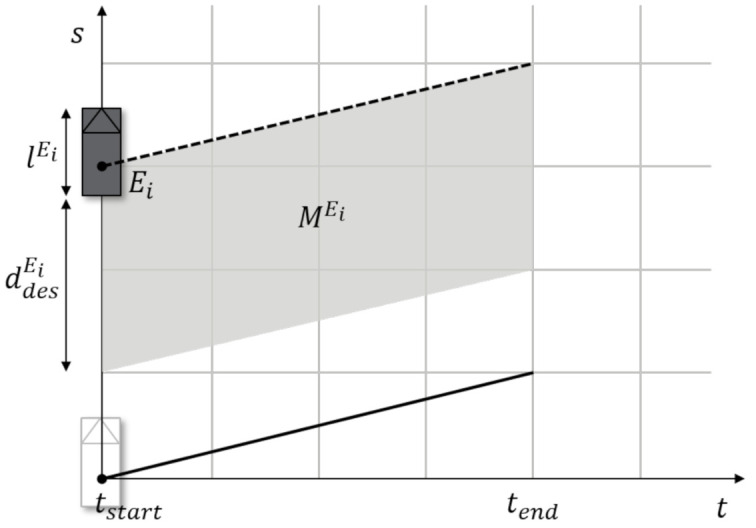
Representation of obstacles in the distance-time domain.

The ego vehicle may be encounter observable and unobservable obstacles such as vehicles and pedestrians. Therefore, the idea is to construct a simple representation for obstacles, which includes the representation of any possible occurrence in the longitudinal direction. An obstacle $E_{i}$ intersects the path at position s(t) at time interval $\tau \in [t_{start},t_{end}]$, then the obstacle $E_{i}$ has a length $l^{E_{i}}$. The obstacle also has a desired following distance $d_{des}^{E_{i}}$, which is defined as a temporal-spatial cost map $M^{E_{i}}$ required to achieve a smooth driving. The cost map $M^{E_{i}}$ is a linear function using the obstacle representation of $E_{i}=\left(s^{E_{i}}\left(t\right),d_{des}^{E_{i}},l^{E_{i}},t_{start},t_{end}\right)$. This map is illustrated in Figure 7. The collision cost can be derived as

(30) $$g_{E}\left(x_{i},x_{i+1},E\right)=\left\{\begin{array}{cc} \infty ,\; & if\exists E_{i}\in E:x_{i+1}\in E_{i}\\ M^{E_{i}}\left(x_{i+1}\right),\; & if\exists E_{i}\in E:x_{i+1}\in E_{i}\\ 0,\; & otherwise \end{array}\right.$$

By applying the reachable set estimation, unobservable obstacles can be regarded as observable obstacles when defined as $E_{i}$. Through the time intersecting the reachable set and the predefined path, we can define $t_{start}^{E_{i}}$ and $t_{end}^{E_{i}}$, and the intersecting distance then describes the position of the obstacle. The desired following distance for the unobservable obstacle can subsequently be calculated in the same manner as for the observable obstacle.

(31) $$d_{des}^{E_{i}}=d_{threshold}+v_{ego}\cdot t_{gap}$$

where $d_{threshold}$ refers to the ideal distance at zero speed, $v_{ego}$ is the current velocity of the ego vehicle, and $t_{gap}$ denotes the ideal time gap for the front obstacle. The linear function for the cost map $M^{E_{i}}$ can then be written as

(32) $$M^{E_{i}}=\left\{\begin{array}{cc} d_{des}^{E_{i}}-d^{E_{i}},\; & ifd^{E_{i}}<d_{des}^{E_{i}}\\ 0,\; & otherwise \end{array}\right.$$

### 5.4. Heuristics

An appropriate and consistent heuristic cost can reduce the computation of A* algorithm. Here, we use Inevitable Collision States (ICSs) [29] as a heuristic cost. An ICS is a state that cannot avoid at least one collision in the future. When a new state is expanded, this state is tested for whether the new state is an ICS. If this is true, i.e., the new state cannot avoid a collision, the remaining heuristic cost $h_{x,i}$ is the maximum collision cost. Through the ICS, the planner enables admissible reactions to upcoming obstacles. In the case of a movement in a one-dimensional direction, the ICS test can be easily done analytically. The ICS test for newly generated state $x_{i}$ can be derived as

(33) $$\forall a\in A\exists E_{i}\in E:\left\{s_{i}+v_{i}\cdot t+\frac{1}{2}a\cdot t^{2}|t\in \left[0\;\infty \right]\right\}\neq \emptyset$$

**Figure 8:**
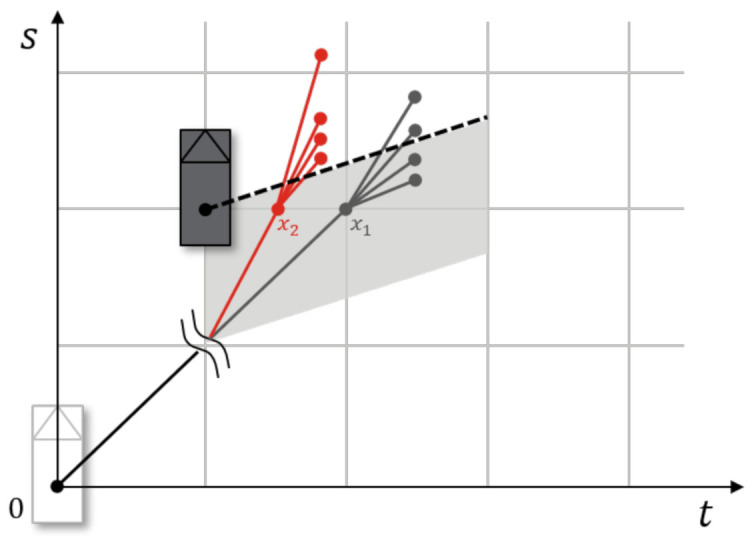
Analytic calculation of the Inevitable Collision State.

Figure 8 presents the concept of an ICS. There are two different initial states $x_{1}$ and $x_{2}$, with $v_{1}<v_{2}$. A collision cannot be avoided for $v_{2}$, whereas the vehicle can avoid the obstacle with $v_{1}$. The heuristic function can be written as

(34) $$h\left(x_{i}\right)=\left\{\begin{array}{cc} \infty ,\; & ifx_{i}\in ICS\\ 0,\; & otherwise \end{array}\right.$$

### 5.5. Goals

The original A* algorithm has a specific goal in the constructed graph. Since we build the graph online, the goal may or may not be reached. Therefore, we set a partial goal in the time domain $t_{G}$. No matter where the vehicle is or its speed, the planner stops finding the speed profile when the time of state $t_{i}$ reaches $t_{G}$.

## 6. Simulation Results

**Figure 9:**
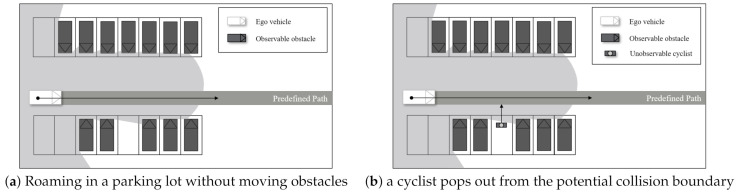
Scenario descriptions.

We evaluated the proposed algorithm using two scenarios, as shown in Figure 9. The first scenario includes the ego vehicle roaming in a parking lot where parked vehicles exist. From this scenario, the proposed method shows the ability to drive safely in limited visibility. The second scenario includes a cyclist that pops out from the potential collision boundary. Through the second scenario, the safety of the proposed algorithm will be proved.

The tests are conducted using a Robot Operating System (ROS) platform [30] in a CARLA simulator [31]. The proposed algorithm was implemented using an i5 Core PC in C++ language. The execution periods of the overall planning process were 100 ms. The total planning horizon was 5 s, and the time step was set to 0.5 s.

### 6.1. Scenario 1: Roaming in a Parking Lot without Obstacles

As first online scenario, roaming in a parking lot is presented. The ego vehicle roams a parking lot with many parked vehicles. The ego vehicle must consider unobservable obstacles that suddenly pops out between the parked vehicles. In other words, the ego vehicle should consider the uncertain prediction of unobservable obstacles which is realized by the reachable set estimation. This uncertainty is incorporated by estimating reachable set in Section 4.

**Figure 10:**
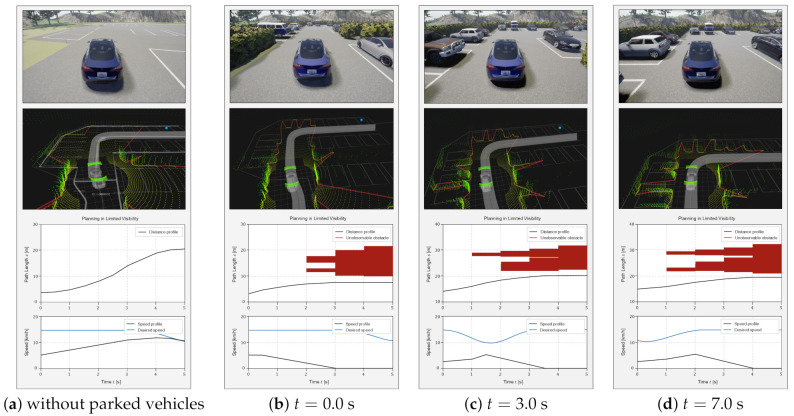
Roaming in a parking lot without obstacles. The upper figure of each time step shows the situation in CARLA. The second figure describes the algorithm in ROS platform. The last figure shows the speed profile at each time step.

In Figure 10a, as a control with the proposed algorithm, the ego vehicle can be seen driving in a parking lot without other parked vehicles. On the other hand, Figure 10 shows the process of roaming in a parking lot. At each figure of Figure 10b–d, the upper figure shows the ego vehicle’s front camera image. The middle figure describes the algorithm details in ROS platform. In this figure, the green points are point cloud and the red lines refers the potential collision boundaries (PCBs). The lower figure shows the estimated reachable set as the red polygon and the speed profile generated by the proposed algorithm.

The ego vehicle generates the speed profile to follow the regulation speed described as the blue line in the lower figure in Figure 10a if the risk of limited visibility does not exist. On the contrary, at t=0 (see Figure 10b), the ego vehicle moves toward an empty parking space without observable obstacles. The ego vehicle tries to generate a speed profile to keep up the regulation speed. However, the ego vehicle cannot speed up until it reaches the regulation speed because it collides with invisible obstacles when speeding up. At that time, the prediction of unobservable obstacles is realized by reachable set estimation as shown in the red polygon in the lower figure of Figure 10b–d. The reachable set is transformed into the distance-time domain with the red polygon in the lower figure. Then, the ego vehicle continuously decelerates until the field of view is large enough to eliminate PCBs. Since the parking lot is not enough to secure visibility due to parked vehicles, the ego vehicle maintains a low speed than the regulation during roaming. (see Figure 10b–d).

### 6.2. Scenario 2: Roaming in a Parking Lot with Obstacles

Same as the first scenario, the ego vehicle roams a parking lot with many parked vehicles. The difference is that a cyclist suddenly pops out from behind a parked vehicle after few second. The ego vehicle cannot detect the cyclist due to the occlusion. This scenario evaluates the model capability when an obstacle pops out from the PCB. In this scenario, an unobservable obstacle is detected at the PCB, at which time it is converted into an observable obstacle. Consequently, the proposed algorithm highlights its scalability for all types of obstacles (unobservable and observable).

**Figure 11:**
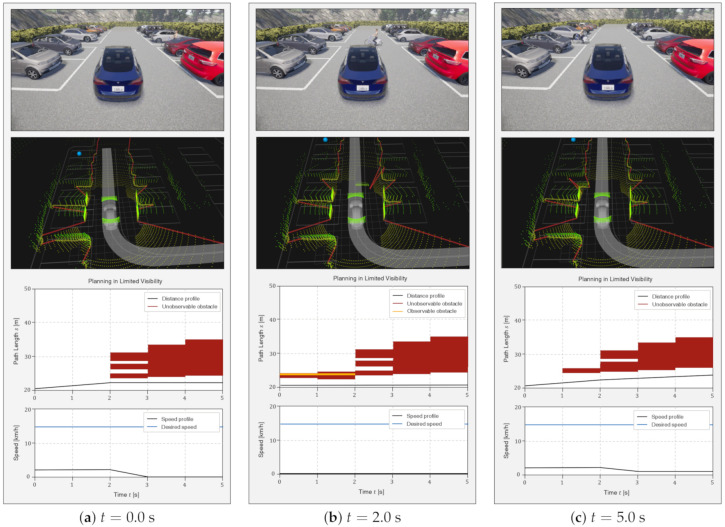
Roaming in a parking lot with obstacles. The upper figure of each time step shows the situation in CARLA. The second figure describes the algorithm in ROS platform. The last figure shows the speed profile at each time step.

Initially, the ego vehicle drives in a parking lot as shown in Figure 11a. Similar to the above simulation, the proposed algorithm tries to decrease the speed slower than the regulation. At the same time, a cyclist is behind the parked vehicle; however, the ego vehicle cannot detect the occluded cyclist. Even though the ego vehicle suddenly encounters the cyclist at t=2 as shown in Figure 11b, the proposed algorithm can stop to avoid a collision with the cyclist since the speed is slow enough as it nears the PCB. The cyclist is described as the yellow polygon in the Figure 11b. The proposed algorithm waits for the cyclist to pass, then increases the speed to arrive at the empty parking space, as shown in Figure 11c. This simulation results confirm that the reachable set estimation is well defined to consider unobservable obstacles occluded by other traffic participants. Moreover, the cost and heuristic functions of the guided A* algorithm are guaranteed to consider both observable and unobservable obstacles.

## 7. Conclusions

This paper presented an algorithm for safe motion planning in condition of limited visibility in a parking lot. The proposed algorithm consists of three steps: Potential Collision Boundary (PCB) Estimation, Reachable Set Estimation-based Motion Prediction, and Motion Planning with A*. To consider visibility limited by occlusions, we defined a PCB to describe the risk of unobservable obstacles. By applying a reachable set estimation as the motion prediction, the PCB is extended to the reachable area once it is determined that an unobservable obstacle exists. Through over-approximations, the reachable set estimation represents the worst-case of unobservable obstacles. We then rewrite the reachable set to include a state for the planning space, such that a simple representation model can convert the reachable set to obstacles in the A* algorithm. In this way, we use the A* algorithm as a motion planner to achieve completeness, optimality, and optimal efficiency. Using this algorithm, we can formulate the cost function to ensure the vehicle follows the desired speed, ensure the vehicle is driving comfortably, and that it avoids collisions. In addition, the Inevitable Collision State (ICS) is applied to the heuristic function to reduce the computational burden.

The simulation results show the ability of the algorithm to generate an optimal speed profile for unobservable obstacles. Through the proposed algorithm, the vehicle could drive in an occluded area with no collisions. Since we enhanced the reachable set by using over-approximations, we could confirm that the planner planned safe speed profiles. The performance is shown via roaming scenarios with occlusions from parked vehicles. All collisions could be prevented while still moving through the parking lot at a sufficient speed. The proposed planner could proactively adjust to unobservable obstacles using one algorithm in an optimized manner, by solving the optimization problem.

Nevertheless, future work will be extended in two ways. On the first hand, the idea is to apply to real driving. The most important thing to apply a planning algorithm to real driving is to handle the uncertainty of input, in this case, the point cloud. If the point cloud measurement is noisy, it causes both the free space and the PCB are also noisy. Then, the planner might mispredict the reachable set of potential collision obstacles. Thus, the speed profile can be noisy in real driving. To overcome this problem, we should develop a PCB tracking algorithm or a fusion algorithm between free spaces from LiDAR and from the camera. Second, we proposed a motion planner that only considers a longitudinal direction. In other words, we solved the problem by either reducing or increasing the speed. However, in more complex situations, such as crossing an intersection in a parking lot, waiting for a parked vehicle, and encountering a car in a narrow alleyway, the planner must change paths laterally. Therefore, we should consider both longitudinal and lateral directions of the trajectory. To consider a lateral direction; however, the complex situation will be divided into a normal state and an inevitable state. A normal state refers to the problem described in Section 3, whereas an inevitable state indicated the complicated situation mentioned above. The different methods should be applied to different types of problems.

## Data Availability

Data sharing not applicable.
